# Trypanosome Diversity in Wildlife Species from the Serengeti and Luangwa Valley Ecosystems

**DOI:** 10.1371/journal.pntd.0001828

**Published:** 2012-10-18

**Authors:** Harriet Auty, Neil E. Anderson, Kim Picozzi, Tiziana Lembo, Joseph Mubanga, Richard Hoare, Robert D. Fyumagwa, Barbara Mable, Louise Hamill, Sarah Cleaveland, Susan C. Welburn

**Affiliations:** 1 Centre for Infectious Diseases, College of Medicine and Veterinary Medicine, The University of Edinburgh, Edinburgh, United Kingdom; 2 Institute for Biodiversity, Animal Health and Comparative Medicine, College of Medicine, Veterinary Medicine and Life Sciences, University of Glasgow, Glasgow, United Kingdom; 3 Tanzania Wildlife Research Institute - Messerli Foundation Wildlife Veterinary Programme, Arusha, Tanzania; 4 Tanzania Wildlife Research Institute - Serengeti Wildlife Research Centre, Arusha, Tanzania; Foundation for Innovative New Diagnostics (FIND), Switzerland

## Abstract

**Background:**

The importance of wildlife as reservoirs of African trypanosomes pathogenic to man and livestock is well recognised. While new species of trypanosomes and their variants have been identified in tsetse populations, our knowledge of trypanosome species that are circulating in wildlife populations and their genetic diversity is limited.

**Methodology/Principal Findings:**

Molecular phylogenetic methods were used to examine the genetic diversity and species composition of trypanosomes circulating in wildlife from two ecosystems that exhibit high host species diversity: the Serengeti in Tanzania and the Luangwa Valley in Zambia. Phylogenetic relationships were assessed by alignment of partial 18S, 5.8S and 28S trypanosomal nuclear ribosomal DNA array sequences within the Trypanosomatidae and using ITS1, 5.8S and ITS2 for more detailed analysis of the *T. vivax* clade. In addition to *Trypanosoma brucei*, *T. congolense*, *T. simiae*, *T. simiae* (Tsavo), *T. godfreyi* and *T. theileri*, three variants of *T. vivax* were identified from three different wildlife species within one ecosystem, including sequences from trypanosomes from a giraffe and a waterbuck that differed from all published sequences and from each other, and did not amplify with conventional primers for *T. vivax*.

**Conclusions/Significance:**

Wildlife carries a wide range of trypanosome species. The failure of the diverse *T. vivax* in this study to amplify with conventional primers suggests that *T. vivax* may have been under-diagnosed in Tanzania. Since conventional species-specific primers may not amplify all trypanosomes of interest, the use of ITS PCR primers followed by sequencing is a valuable approach to investigate diversity of trypanosome infections in wildlife; amplification of sequences outside the *T. brucei* clade raises concerns regarding ITS primer specificity for wildlife samples if sequence confirmation is not also undertaken.

## Author Summary

The trypanosomes include a number of species that cause disease in livestock. In recent years, several trypanosomes have been identified which do not fit into the classic trypanosome classification system. However, previous work has focused on trypanosomes identified in the tsetse vector, with little information available on trypanosomes found in their natural hosts, wildlife. We studied trypanosome sequences from wildlife in Serengeti National Park in Tanzania and the Luangwa Valley in Zambia and found a number of trypanosome species pathogenic to livestock were circulating in these areas. For *Trypanosoma vivax*, one of the causes of trypanosomiasis in cattle, variants were identified in giraffe and waterbuck that were different from all published sequences and from each other. These variants did not test positive with the molecular tests usually used to identify *T. vivax* suggesting that *T. vivax* may often be under-diagnosed in Tanzania. The trypanosome classification system is facing challenges as molecular data are incorporated into a system that historically was based on factors such as morphology, host range and geographical distribution.

## Introduction

The African trypanosomes include a number of species of importance for human and livestock health (Table 1). Trypanosome classification was for many decades based on morphology, host range, distribution and pathogenicity but accumulating molecular evidence shows this is an oversimplification. Phylogenetic data have indicated the existence of previously unidentified trypanosome species, subspecies and variants [1]. Most of the ‘new’ trypanosomes identified have come from investigations into trypanosomes found in tsetse flies. Identification of *T. simiae* Tsavo followed the failure of a trypanosome to hybridise with existing DNA probes [2] and similarly *T. godfreyi* was described when isoenzyme and DNA analysis indicated a trypanosome that differed from previously recognised species found in *Glossina morsitans submorsitans* in The Gambia [3]. Investigations of tsetse populations in Tanzania indicated a parasite that failed to amplify with existing PCR primers and led to the designation ‘*T. godfreyi*-like’ [4] and ‘*T. brucei*-like’ [5] parasites in tsetse flies.

**Table 1:** Summary of the host range and pathogenicity of the Salivarian trypanosomes.

Species	Description
*T. brucei*	In east and southern Africa *T. brucei rhodesiense* causes human African trypanosomiasis. *T. brucei brucei* is pathogenic to camels, horses and dogs. *T. b. brucei* and *T. b. rhodesiense* are also found in cattle, sheep, goats and pigs but cause mild or no clinical disease, and in a wide range of wildlife species. *T. b. gambiense* causes human African trypanosomiasis in west and central Africa and has also been reported in pigs and several wildlife species.
*T. congolense*	Most important as a pathogen of cattle but can also cause disease in other species, including sheep, goats, pigs, horses and dogs [53], [54]. Has been identified in a wide range of wildlife species, including Bovidae and Suidae [7], [26], [28]. Three groups – savannah, riverine forest and Kenya coast or Kilifi [42].
*T. vivax*	Most important as a pathogen of cattle but also causes disease in sheep, goats, horses and camels. Found in a wide range of wildlife species including Bovidae and Suidae [28], [55].
*T. simiae*	Causes acute, fatal disease in pigs [53], and mild disease in sheep and goats. Predominantly associated with wild suids [26], [56]. Subspecies *T. simiae* Tsavo isolated from tsetse [2], causes mild disease in domestic pigs experimentally [57] and has been reported in warthogs [26].
*T. godfreyi*	Only isolated from tsetse but causes chronic, occasionally fatal disease in pigs experimentally [3].

These investigations of ‘novel’ trypanosomes in tsetse flies do not provide information on the life of trypanosomes within their vertebrate hosts but do provide a quick method of identifying potential new agents within a system. To identify trypanosome host ranges and diversity it is essential to study trypanosomes that are circulating within and between wildlife (and other) hosts. As wildlife can act as reservoirs of trypanosomes pathogenic to both humans and livestock [6], [7], [8], understanding trypanosomes circulating in wildlife populations has implications for control of diseases of economic and public health importance and is critical information for agencies following a One Health approach to disease management [9].

Limited information exists on the trypanosome species present in different wildlife species or their genetic diversity. Early studies on wildlife relied on microscopy, for example [7], [10], that is unreliable for trypanosome species identification and for differentiating within subgenera (between *T. congolense*, *T. simiae* and *T. godfreyi*) or if mixed infections are present. Microscopy also has a low sensitivity [11], particularly problematic in wildlife species, which often show low parasitaemia [12]. The relatively recent description of *T. godfreyi* and *T. simiae* Tsavo means that although these trypanosomes appear widespread in certain tsetse populations [4], [13], [14], their natural hosts are not well described. The logistical difficulties of obtaining samples from free-ranging species has limited studies on wildlife, with most phylogenetic information limited to single animals [15]. Therefore, despite continuing discussions on the taxonomic implications of new species, subspecies and groups of trypanosomes identified in tsetse populations [1], little progress has yet been made in exploring trypanosome diversity in the wildlife hosts where these trypanosomes evolved.

A suite of molecular tools have been developed to identify trypanosomes, both in tsetse and in vertebrate hosts [16], [17]. PCR primers which target species-specific repetitive satellite DNA sequences have been described for identification of *T. brucei* sensu lato, *T. congolense* (savannah, forest and Kilifi groups), *T. vivax, T. simiae, T. simiae Tsavo and T. godfreyi* [2], [18], [19], [20], [21]. For *T. vivax*, the target sequence is not present in all isolates, particularly in East Africa; additional primers have been developed based on a sequence from a gene encoding a differentially expressed protein captured in an antigen detection enzyme-linked immunosorbent assay, thought to be found in all *T. vivax* [22]. The prevalence of *T. vivax* in tsetse populations in Tanzania was found to be higher using these primers, compared with those based on satellite DNA sequences [4].

Species-specific primers amplify only the target species, and will not amplify unidentified or diverse trypanosomes that do not carry the target sequence. Primers which target the internal transcribed spacer (ITS) regions of ribosomal DNA rely on species-specific differences in sequence length to differentiate trypanosome species [23], [24], [25]. These primer sites are well conserved across trypanosome species; even sequences from diverse or previously unidentified trypanosomes are likely to be amplified - particularly important in identifying trypanosomes in wildlife hosts.

Serengeti National Park, Tanzania and Luangwa Valley, Zambia comprise areas of high wildlife density and diversity. In addition, around both of these ecosystems, rural livelihoods are dependent on small-scale livestock production, including cattle, sheep, goats and pigs. The importance of trypanosomiasis in livestock in these areas is well recognised, with prevalence of 5% for *T. congolense*, 0.6% for *T. vivax* and 6% for *T. brucei* (using species-specific primers) in cattle around Serengeti [26], and prevalence of 74% for *T. congolens*e, 23% for *T. vivax* and 2% for *T. brucei* (using ITS primers) in cattle in Luangwa Valley [27].

In this study we used ITS primers [23] to amplify partial 18S, ITS1, 5.8S, ITS2 and partial 28S regions of ribosomal DNA to identify trypanosome species circulating in two wildlife-rich ecosystems. Clonal sequence analysis was carried out to confirm the identity of trypanosomes found and to explore the phylogenetic relationships among identified sequences.

## Materials and Methods

### Field sample collection

Blood samples were collected from a range of wildlife species in Serengeti National Park, Tanzania between 2002 and 2007, and Luangwa Valley, Zambia between 2005 and 2007. In Tanzania samples were collected from animals found dead, or animals immobilised for conservation management or disease surveillance purposes. In animals found dead, blood samples were collected from the heart if a post mortem examination was conducted and from larger peripheral veins or blood pools in the carcase if no post mortem examination was carried out. The cause of death was not routinely established but included kills by predators and road traffic accidents. Time between death and sampling was estimated not to exceed six hours. In Zambia, samples were collected from animals immobilised as part of routine conservation management activities or from animals harvested as part of commercial safari hunting operations in game management areas. Further details have been published previously [28]. Whole blood samples were preserved on FTA classic cards (Whatman Biosciences, Cambridge, UK).

### Ethics statement

This study utilised blood samples collected from wild animals. In Tanzania samples were collected opportunistically from animals found dead, or immobilised for other reasons such as to put on radio collars. Additional samples were collected from warthogs which were immobilised to collect blood samples for trypanosome surveillance. Animals were released unharmed after sampling. All activities were approved by the Tanzania Wildlife Research Institute, Tanzania National Parks and Tanzania Commission for Science and Technology (permit numbers 2005-102-CC-2005-07, 2006-143-ER-2005-07, 2007-163-ER-2005-07). In Zambia samples were collected from animals that had already been shot as part of commercial safari hunting activities under a strictly licensed quota system managed by the Zambian Wildlife Authority. These animals were not shot for the purpose of this study. Additional samples were also collected from animals captured and released unharmed as part of a translocation exercise for the Zambia/Malawi Transfrontier Conservation Area. All activities in protected areas were fully approved by the Zambian Wildlife Authority (permit numbers 316295 and 323947). All sampling protocols were approved by the Zambian Wildlife Authority and the Zambian Department of Veterinary and Livestock Development. All sampling protocols adhered to relevant national guidelines (from Tanzania Wildlife Research Institute and Zambia Wildlife Authority) for handling and sampling free ranging wildlife. For all samples the relevant export and import licences were obtained, including CITES permits for samples from animals on CITES appendices 1 and 2.

### Sample preparation and PCR

Five discs per sample were cut from FTA cards using a 3 mm diameter Harris Micro Punch tool. Between each sample, 2 punches were taken from clean filter paper, to prevent cross-contamination. Discs were prepared for analysis using the following protocol: two washes of 15 minutes each with FTA purification reagent (Whatman Biosciences, Cambridge, UK), followed by two washes of 15 minutes each with TE buffer (Sigma Aldrich, Dorset, UK). Discs were dried at room temperature for 90 minutes, then incubated with 5% chelex solution at 90°C for 30 minutes to elute DNA from the card [29].

The ITS primers described by Cox et al. [23] were used to amplify the partial 18S, ITS1, 5.8S, ITS2 and partial 28S regions (Table 2). PCR was carried out in 25 µl reaction volumes, containing 10 mM Tris-HCl pH 9.0, 1.5 mM MgCl2, 50 mM KCl, 0.1% TritonX-100 and 0.01% (w/v) stabiliser (all combined in SuperTaq PCR buffer, HT Biotechnologies, Cambridge, UK), 2 µM of each outside primer ITS1 and ITS2, 1 mM total dNTPs, 1.25 Units of Biotaq (Bioline Ltd, London, UK), and 1 µl of eluted DNA. The second round reaction contained 1 µl of first round product, and used internal primers ITS3 and ITS4. Each PCR batch included genomic DNA positive controls, one negative disc and one water negative control. Thermal cycling was carried out in a DNA Engine DYAD Peltier thermal cycler. PCR products were run on 1.5% (w/v) agarose gels at 100 V, stained with ethidium bromide and visualised under an ultraviolet transilluminator.

**Table 2:** PCR primers and cycling conditions.

PCR	Primer Sequence	Product size
ITS [23]	ITS 1: 5′ - GATTACGTCCCTGCCATTTG- 3′	*T. congolense* 1408–1501 bp
	ITS 2: 5′ - TTGTTCGCTATCGGTCTTCC- 3′	*T. brucei* 1215 bp
	ITS 3: 5′ - GGAAGCAAAAGTCGTAACAAGG- 3′	*T. theileri* 998 bp
	ITS 4: 5′ - TGTTTTCTTTTCCTCCGCTG- 3′	*T. simiae* Tsavo 951 bp
		*T. simiae* 847 bp
	Cycling Conditions: 95°C for 7 min, 35 cycles: 94°C for 60 sec, 55°C for 60 sec, 72°C for 120 sec	*T. vivax* 620 bp
*T. godfreyi* [20]	DGG1: 5′-CTGAGGCTGAACAGCGACTC-3′	373 bp
	DGG2: 5′-GGCGTATTGGCATAGCGTAC-3′	
	Cycling conditions: 92°C for 1 min, 30 cycles: 92°C for 30 sec, 60°C for 60 sec, 72°C for 30 sec	
*T. vivax* [22]	ILO1264: 5′-CAGCTCGGCGAAGGCCACTTCGCTGGGGTG-3′	400 bp
	ILO1265: 5′-TCGCTACCACAGTCGCAATCGTCGTCTCAAGG-3′	
	Cycling conditions: 30 cycles: 94°C for 60 sec, 55°C for 120 sec, 72°C for 120 sec.	

### Clonal sequence generation

ITS primers generate PCR products of varying length, depending on the trypanosome species, subspecies or group [23], listed in Table 2. In this study, ITS PCR results showed band sizes between 550 and 1000 bp which were not consistent with the expected sequence lengths. Bands were selected from this size range for sequencing. In addition, bands were sequenced from two samples that were of the size expected for *T. brucei* and *T. congolense*, to confirm the identity of these bands. DNA was extracted from selected bands in agarose gels using a Qiagen MinElute DNA extraction kit (Qiagen, Crawley, UK) following manufacturer's protocols. Cloning was carried out using a Qiagen PCR Cloning kit. The ligation-reaction mixture contained 4 µl of purified PCR product, 1 µl of pDrive cloning vector (50 ng/µl) and 5 µl of distilled water and was incubated at 4°C for two hours, and otherwise followed manufacturer's protocols. Plasmid DNA was purified using the Qiaprep Spin Miniprep kit and the eluted DNA was submitted for sequencing (GATC Biotech, Germany) with M13 forward and reverse primers. One clone was submitted for each selected band.

### Sequence analysis

Initial sequence inspection and cleaning was conducted in Bioedit [30]. Sequences were identified by BLAST search (NCBI Blastn). Sequence similarity was also assessed by shared percent identity over the whole sequence: (i) between sequences generated in this study and available reference sequences; and (ii) between sequences generated in this study identified as the same species or group. For *T. godfreyi*, the only existing sequence in Genbank for comparison covered ITS1 only (130 bp). For *T. vivax*, existing sequences covered ITS1, 5.8S and ITS2 only (534 bp). Blast searches and shared identity assessment were therefore conducted over these reduced sequence lengths.

**Figure 1:**
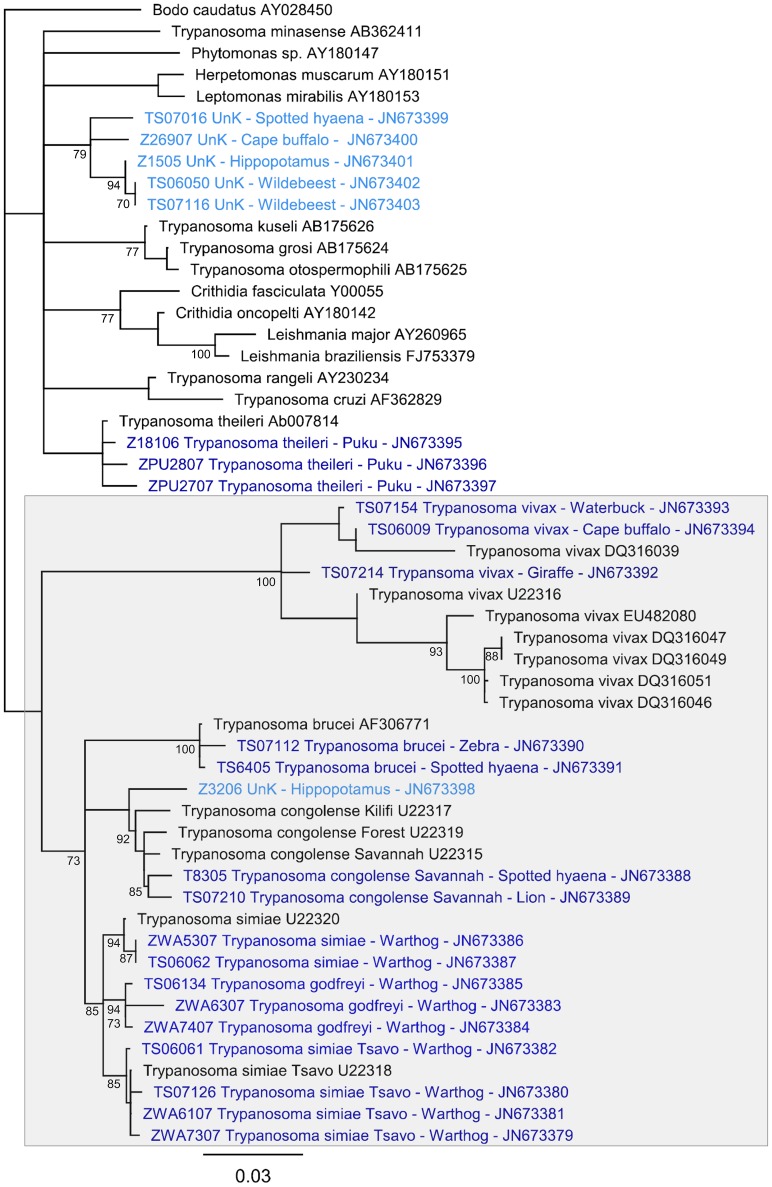
Neighbour-joining tree based on partial 18S, 5.8S and partial 28S trypanosomatid sequences. *Bodo caudatus* was included as an outgroup. Bootstrap values are shown where support is >70%. Sequences generated in this study are shown in blue (identified sequences in dark blue, unidentified sequences in light blue), and labelled with sample identity; pathogen species (UnK if unknown); host species; Genbank ID. Other sequences were retrieved from Genbank and are shown in black, and are labelled with pathogen species and Genbank ID. *T. brucei* clade indicated in grey box.

Phylogenetic analyses were conducted to infer the relationships of sequences generated in this study with other trypanosomes. The partial 18S, 5.8S and partial 28S sequences were aligned using the ClustalW [31] accessory application in Bioedit, followed by visual optimisation (it was not possible to align the ITS1 and ITS2 regions across all variants found, due to their hypervariability). In addition to all sequences generated in this study, included in the alignment were sequences listed in Genbank for this region within the *T. brucei* clade; we use *T. brucei* clade to refer to the clade which includes *T. brucei*, *T. congolense*, *T. simiae*, *T. godfreyi* and *T. vivax* and related subspecies and groups, as by [32]: only one sequence each was available for *T. congolense* savannah, forest and Kilifi, *T. simiae*, *T. simiae* Tsavo and *T. godfreyi*; for *T. brucei* one representative sequence was included; for *T. vivax*, one clone of each of the seven available published sequences was used. In addition, published sequences from outside the *T. brucei* clade but within the Trypanosomatidae were included to help identify more diverse sequences. Accession numbers for all reference sequences are included in Figure 1. A neighbour-joining tree was constructed using Geneious [33] under a Hasegawa-Kishino-Yano (HKY) [34] model of substitution. *Bodo caudatus* was included as an outgroup; *B. caudatus* is a member of the Bodonidae, another kinetoplastid family, and has been shown to be a valid outgroup for trypanosomatids [32]. Confidence in branching relationships was assessed using bootstrap re-sampling over 1000 replicates. Using the same alignment, trees were also constructed in PAUP * 4.0 [35] using minimum evolution and maximum likelihood optimality criteria, both with an HKY model of substitution and default settings for the heuristic searches conducted.

**Figure 2:**
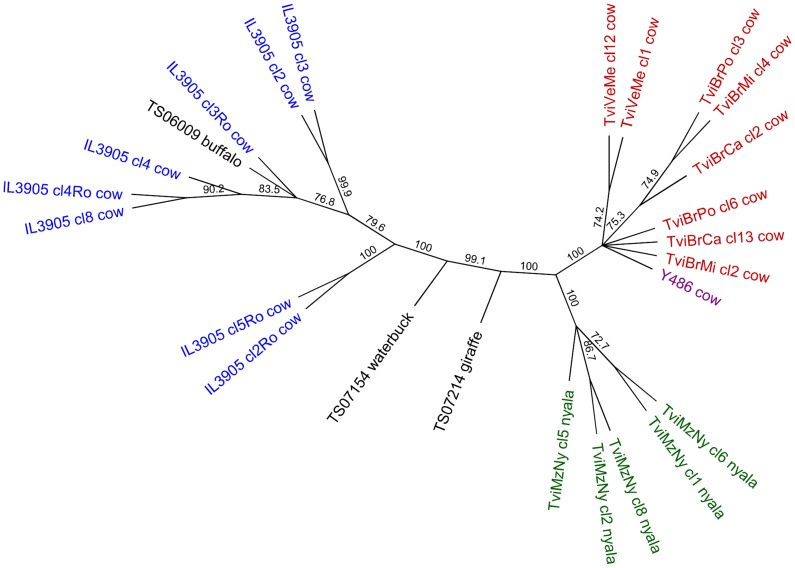
Unrooted neighbour-joining dendrogram of ITS1, 5.8S and ITS2 sequences for *Trypanosoma vivax*. Bootstrap values are shown at nodes with >70% support. Sequences generated in this study shown in black; other sequences retrieved from Genbank and listed in Table 3. Sequence locations are shown by colour: Tanzania (this study, black); Kenya (blue); Mozambique (green); Nigeria (purple); South America (red). Host species from which sequence was amplified are indicated. Accession numbers for reference sequences are: IL3905 cl8, Genbank ID:DQ316040; IL3905 cl4Ro, DQ316043; IL3905 cl4, DQ316039; IL3905 cl3Ro, DQ316042; IL3905 cl2Ro, DQ316041; IL3905 cl5Ro, DQ316044; IL3905 cl2 DQ316037; IL3905 cl3, DQ316038; TviBrMi cl4, DQ316048; TviBrPo cl13, DQ316049; TviBrCa cl2, DQ316045; Y485, U22316; TviBrMi cl2, DQ316047; TviVeMe cl1, DQ316051; TviVeMe cl12, DQ316052; TviBrCa cl13, DQ316046; TviBrPo cl6, DQ316050; TviMzNy cl5, EU482080; TviMzNy cl2, EU482079; TviMzNy cl8, EU482082; TviMzNy cl1, EU482078; TviMzNy cl6, EU482081.

Blast results suggested that *T. vivax* sequences generated in this study did not closely match existing sequences. To assess the phylogenetic relationships within the *T. vivax* clade, ITS1, 5.8S and ITS2 sequences were aligned for all sequences clustering in this group, together with all *T. vivax* sequences available in Genbank for this region, and an unrooted neighbour-joining tree constructed using a HKY model of substitution in Geneious, with bootstrap values calculated for 1000 replicates. Accession numbers for all *T. vivax* reference sequences are listed in Figure 2.

### Additional PCR analysis

Sequences which were clustered with *T. vivax* were also tested with *T. vivax*-specific primers to establish whether these trypanosomes would be detected by conventional species-specific primers. The primers described by Masake et al. [22] were used that have been shown to be most appropriate for *T. vivax* in Tanzania [4]. PCRs were performed in triplicate on eluted DNA, following the published protocol and cycling conditions [22] (Table 2).

For *T. godfreyi*, the only reference sequence available for the ITS region covered only ITS1. Therefore, for two samples where *T. godfreyi* was tentatively identified on the basis of ITS1 similarity, confirmatory *T. godfreyi*-specific PCRs were also conducted, using the primers and conditions listed in Table 2 [20].

## Results

### Blast searches

Thirty-two new ribosomal DNA array sequences were generated from wildlife samples. Close matches were obtained to existing Genbank trypanosome sequences for 19 of the sequences generated in this study (Table 3). Sequences identified from zebra and spotted hyena closely matched existing sequences from *T. brucei* s.l. and sequences identified from spotted hyena and lion closely matched *T. congolense* savannah. Sequences TS07126, TS06061, ZWA7307 and ZWA6107 all obtained from warthogs and sized between 967 and 972 bp, shared 90–91% identity with *T. simiae* Tsavo (U22318) over the whole sequenced region. *T. simiae* Tsavo sequences from Serengeti (n = 2) and Luangwa (n = 2) were very similar, sharing 96–98% identity over the whole sequence length. TS06062 and ZWA5307 from warthog most closely matched *T. simiae*, sharing 86% identity with the one available reference sequence U22320. Sequences from warthogs in Serengeti and Luangwa shared 97% identity with one another. Sequences found in warthogs from both Serengeti (TS06134) and Luangwa (ZWA6307, ZWA7407) most closely matched *T. godfreyi*, although only the ITS1 sequence is available in Genbank for this species (130 bp, AY661891). ZPU2807, ZPU2707 and Z18106, all from puku, were most similar to *T. theileri*. These sequences shared 79–80% identity with AB007814, identified from a cow, but were approximately 70 bp shorter than the expected sequence length [23]. They were very similar to each other, sharing 98–99% identity.

**Table 3:** Identification of sequences.

Sample Number	Location	Host species	Sequence length (bp)	Closest match on BLAST - Genbank ID, species, sequence similarity %	
TS07210	Serengeti	Lion	1406	U22315 *T. congolense*	97
T8305	Serengeti	Spotted hyaena	1419	U22315 *T. congolense*	95
T6405	Serengeti	Spotted hyaena	1220	XO5682 *T. brucei*	99
TS07112	Serengeti	Zebra	1207	AC159414 *T. brucei*	97
ZPU2807	Luangwa	Puku	930	AB007814 *T. theileri*	79
ZPU2707	Luangwa	Puku	931	AB007814 *T. theileri*	79
Z18106	Luangwa	Puku	930	AB007814 *T. theileri*	79
TS07126	Serengeti	Warthog	967	U22318 *T. simiae* Tsavo	90
TS06061	Serengeti	Warthog	968	U22318 *T. simiae* Tsavo	91
ZWA7307	Luangwa	Warthog	974	U22318 *T. simiae* Tsavo	90
ZWA6107	Luangwa	Warthog	968	U22318 *T. simiae* Tsavo	91
TS06062	Serengeti	Warthog	879	U22320 *T. simiae*	86
ZWA5307	Luangwa	Warthog	874	U22320 *T. simiae*	86
TS06134	Serengeti	Warthog	650	AY661891 *T. godfreyi*	88^a^
ZWA6307	Luangwa	Warthog	651	AY661891 *T. godfreyi*	88^a^
ZWA7407	Luangwa	Warthog	648	AY661891 *T. godfreyi*	85^a^
TS06009	Serengeti	Cape buffalo	654	DQ316043 *T. vivax*	97^a^
TS07154	Serengeti	Waterbuck	596	DQ316043 *T. vivax*	81^a^
TS07214	Serengeti	Giraffe	594	DQ316041 *T. vivax*	79^a^
**Unidentified or non-trypanosomal sequences**					
Z1505	Luangwa	Hippopotamus	809	No match	
TS06050	Serengeti	Wildebeest	823	No match	
Z3206	Luangwa	Hippopotamus	840	No match	
TS07116	Serengeti	Wildebeest	852	No match	
Z26907	Luangwa	Cape buffalo	1042	No match	
TS07016	Serengeti	Spotted hyaena	1055	No match	
TS07118	Serengeti	Thomson's gazelle	646	No match	
Z9506	Luangwa	Leopard	663	No match	
Z16006	Luangwa	Impala	713	No match	
Z1605	Luangwa	Lion	847	No match	
ZE4107	Luangwa	Zebra	766	EU400587 *Malassezia restricta*	98
Z27907	Luangwa	Buffalo	771	GU370752 Uncultured fungus	98
Z18706	Luangwa	Waterbuck	888	AY028447 *Dimastigella trypaniformis*	82

Blast search results for sequences in this study and sequence similarity with nearest matches (over whole sequence length unless specified).

a Reference sequence only available for part of sequence (AY661891 130 bp; DQ316043 534 bp).

Lion *Panthera leo*; spotted hyaena *Crocuta crocuta*; zebra *Equus burchelli*; puku *Kobus vardonii*; warthog *Phacochoerus africanus*; Cape buffalo *Syncerus caffer*; waterbuck *Kobus ellipsiprymnus*; giraffe *Giraffa camelopardalis*; hippopotamus *Hippopotamus amphibius*; wildebeest *Connochaetes taurinus*; Thomson's gazelle *Gazella thomsoni*; leopard *Panthera pardus*; impala *Aepyceros melampus*.

Ten sequences from zebra, buffalo and waterbuck that were identified did not closely match any existing sequences (see Table 3). Three showed alignment to non-trypanosomatid organisms, *Dimastigella trypanoformis*, *Malassezia restricta* and uncultured fungus.

### Alignment of partial 18S, 5.8S and partial 28S

An alignment of partial 18S, 5.8S and partial 28S sequences (341 characters; 209 for *T. vivax* sequences) was used to reconstruct phylogenetic trees using neighbour joining, minimum evolution and maximum likelihood methods. Regardless of which method was used, sequences from this study clustered with the same reference sequences; the neighbour joining tree is presented (Figure 1).

Sequences identified as *T. brucei*, *T. congolense*, *T. simiae*, and *T. simiae* Tsavo each formed strongly supported groups with the relevant reference sequences (bootstrap values 100, 92, 94, 85 respectively). The sequences tentatively identified as *T. godfreyi* clustered close to *T. simiae* and *T. simiae* Tsavo, as would be expected for *T. godfreyi*. All *T. vivax* sequences, including the three identified in this study, formed a separate clade with 100% bootstrap support, which sat on the periphery of the *T. brucei* clade, as is usually found for *T. vivax* [15], [32].

The thirteen sequences that did not match any existing trypanosome sequence can be considered in three groups: (i) Z3206 from a hippopotamus consistently clustered close to *T. congolense* and *T. brucei* but the exact location was not well resolved; (ii) samples Z26907 (buffalo), TS07016 (spotted hyaena), Z1505 (hippopotamus), TS06050 (wildebeest) and TS07116 (wildebeest) formed a separate group with good boot strap support (79%). This group consistently sat outside the *T. brucei* clade but within the Trypanosomatidae, but the resolution was not sufficient to further identify these sequences; (iii) a third group of sequences consistently sat outside the Trypanosomatidae and included sequences matching other organisms such as *Dimastigella* and *Malassezia*: TS07118 (Thomson's gazelle), Z9506 (leopard), Z16006 (impala), ZE4107 (zebra), Z27907 (buffalo), Z1605 (lion) and Z18706 (waterbuck) (not included in Figure 1).

### Alignment of ITS1, 5.8S and ITS2 for *T. vivax*

Based on the alignment of the complete ITS region for the three *T. vivax* sequences generated in this study with published *T. vivax* sequences, TS06009 from a buffalo was similar to the only available East African reference sequence (IL3905), isolated from a cow in Kenya [36] (Figure 2). Sequences from a waterbuck (TS07154) and giraffe (TS07210), although clearly clustering with *T. vivax*, differed from all existing sequences, including sequences from Kenya (IL3905) and Mozambique (TviMzNy) (Figure 2).

### Additional PCR analysis

Two out of three of the samples identified as *T. godfreyi* were tested with *T. godfreyi* -specific primers and tested positive. The *T. vivax* sequences identified in this study all tested negative with conventional *T. vivax*-specific primers.

## Discussion

Clonal sequence analysis of ITS PCR products from blood samples collected from wildlife species in Serengeti, Tanzania and Luangwa Valley, Zambia, identified a number of trypanosome species, including *T. congolense*, *T. brucei*, *T simiae*, *T. simiae* Tsavo, *T. godfreyi*, *T. vivax* and *T. theileri*, and revealed new diversity within the *T. vivax* clade.

### Phylogenetic trees

Trees were constructed using an alignment of (i) partial 18S, 5.8S and partial 28S sequences and (ii) ITS1, 5.8S and ITS2 for *T. vivax*. The resolution gained from the alignment of 18S, 5.8S and partial 28S sequences was not sufficient to accurately place all clades outside the *T. brucei* clade in relation to one another; however, the aim of this study was to identify sequences rather than obtain perfect resolution of complex phylogenies, which has been well covered by other authors [32], [37].

### Wildlife hosts of *T. godfreyi*, *T. simiae* and *T. simiae* Tsavo

Identification of *T. godfreyi* and *T. simiae* Tsavo in warthogs confirmed suids as hosts of these species. *T. godfreyi* was identified as a new species when found in tsetse [3] and has since been found to be widespread in tsetse populations [4], [13]. Experimental infection of domestic pigs resulted in chronic disease and it was hypothesized that *T. godfreyi* may naturally circulate in warthogs, but we believe this is the first time that *T. godfreyi* has been confirmed in wild suids. *T. simiae* Tsavo was first identified in tsetse in Tsavo National Park, Kenya [2], and was later confirmed as a sub-group of *T. simiae*, rather than *T. congolense* as had first been thought [38], [39], [40]. Experimentally, *T. simiae* Tsavo has only been found to infect pigs; whether warthogs represent the only wild host of these trypanosomes remains unknown.

*T. simiae*, *T. simiae* Tsavo and *T. godfreyi* sequences showed remarkable similarity between Serengeti in Tanzania and Luangwa Valley, Zambia, despite differing from existing sequences. While small differences from the existing sequences could have been explained by errors from the PCR and sequencing processes (given that only one clone was sequenced per sample), the consistency observed between sequences from the two areas precludes this as an explanation. It is interesting to note that all published sequences were identified from tsetse [14], [41]; whether the differences between our sequences and published sequences reflect identification of different strains or an artefact of isolates from tsetse with subsequent rodent passage is unclear.

### Trypanosome classification

*T. simiae*, *T. simiae* Tsavo and *T. godfreyi* are closely related genetically, as well as sharing characteristics of morphology, development in tsetse and host range. *T. godfreyi* was classified as a new species predominantly on the basis of isoenzyme analysis; it was argued that *T. godfreyi* was as genetically and phenotypically distinct from *T. simiae* and *T. congolense* as they were from each other [3]. However, in this study *T. godfreyi* was not notably more different from *T. simiae* than *T. simiae* Tsavo was (nine nucleotide differences between *T. simiae* and *T. godfreyi* compared to six between *T. simiae* and *T. simiae* Tsavo on the alignment of partial 18S, 5.8S and partial 28S) which is consistent with other phylogenetic analyses [4], [40]. In contrast, variants of *T. congolense*, which also show considerable genetic variation, have been classified into distinctive groups – savannah, riverine forest and Kenya coast or Kilifi [42] and it has recently been suggested that variants of *T. vivax* should be grouped into types A, B and C [43] on the basis of genetic differences. Clearly, incorporating genetic data into historic taxonomic classifications is not straightforward, but a more consistent approach is needed. The nomenclature suggested by Adams et al. [43] of naming groups A, B and C should be used more widely as the geographical nomenclature used in the past to name trypanosomes can be misleading: *T. simiae* Tsavo was named after the location of its first identification in Kenya [2] but has since been identified in other areas including Tanzania and Uganda [4], [13], [44]; the subgroups of *T. congolense* have all been found in multiple locations and ecosystems, often with multiple subgroups in one location [4], [45].

### *T. vivax* diversity

We identified three variants of *T. vivax* in three different wildlife species. TS06009 found in this study from a buffalo in Serengeti closely matched sequence IL3905 from a cow in Kenya. However, sequences from a giraffe (TS07214) and a waterbuck (TS07154), whilst clearly within the *T. vivax* clade, were divergent from all existing sequences. Phylogenetic analysis of *T. vivax* previously indicated that whilst isolates from West Africa and South America form a homogeneous lineage, sequences identified from East Africa are both different from the West African and South American sequences and are more diverse [15], [36]. This is consistent with differences between East and West African isolates in clinical presentation, morphology and molecular characteristics [22], [46], [47], [48]. *T. vivax* found in tsetse in Tanzania [4] and *T. vivax* identified in nyala antelope in Mozambique have previously been shown to differ from all other sequences on phylogenetic analysis, including an East African *T. vivax* from Kenya, whilst still clustering in the *T. vivax* clade [15].

TS07154 and TS07214 are distinct from existing sequences including the sequence identified from a nyala in Mozambique (TviMzNy); no sequence was available for the ITS region from the Tanzanian isolate identified by Malele et al. [4]. The high diversity observed here within the *T. vivax* clade echoes findings in *G. pallidipes* and *G. swynnertoni* in Tanzania of two diverse *T. vivax* genotypes [43]. Adams et al. (2010b) term these *T. vivax* A and B, with group C comprising West African and South American *T. vivax* sequences.

The three samples that contained *T. vivax* sequences in this study did not test positive on PCR with species-specific primers for *T. vivax*. It is known that *T. vivax* primers based on a satellite DNA monomer [21] do not amplify all East African *T. vivax*. However, the primers used here target a sequence thought to be present in all *T. vivax* [22], shown previously to be the most appropriate for identification of *T. vivax* in Tanzania [4]. The prevalence of *T. vivax* detected using species-specific primers in other studies in Tanzania has been low; for example, the prevalence of *T. vivax* in cattle around Serengeti National Park was found to be 0.6% using the Masake primers that were also used in this study [26]. If *T. vivax*-specific primers are not detecting *T. vivax* strains circulating in Tanzania, the true prevalence may be much higher and since *T. vivax* is an important livestock pathogen, further work is required to determine the true prevalence.

Although this study looked at only a small number of sequences, analysis of several sequences from different wildlife species in one location provides an opportunity to start exploring reasons for the diversity of the Tanzanian *T. vivax* sequences. Up to now, explanations for differences between *T. vivax* isolates have considered geographical location, with clear differences between isolates from West Africa and South America versus East Africa [36]. The identification of three distinct *T. vivax* sequences in the same ecosystem indicates that the existence of different isolates cannot be explained by geographical variation alone. The possibility of strains specific to different wildlife host species cannot be ruled out. Host-specific strains of *T. theileri* have been identified in cattle and water buffalo within the same geographical areas [49], and selective tsetse feeding may provide an opportunity for host parasite co-evolution [50], [51]. In this study, *T. vivax* from a buffalo matched a sequence from a cow in Kenya, whilst sequences from giraffe and waterbuck differed from existing sequences. Buffalo and domestic cattle are both Bovinae and may be more likely to share more similar pathogen susceptibility than cattle would share with giraffe or waterbuck. However, more information is needed to test these hypotheses; a study to generate more information on trypanosomes and host sharing between buffalo and cattle is currently underway. Further characterisation of *T. vivax* in wildlife is clearly necessary, particularly to look at the circulation of strains within and between wildlife host species, and any relevance this may have for transmission to, and pathogenicity in, livestock.

Further investigation of trypanosome infections in wildlife hosts relies on characterisation of the interactions of vector, host and trypanosome. In particular, incorporating information on the prevalence of trypanosome species in different wildlife hosts and tsetse blood meal data will help to elucidate the relative roles of host immune response compared to tsetse feeding patterns and further manuscripts are in preparation on this subject.

### Unidentified or non-trypanosomatid sequences

Thirteen sequences did not closely match any existing sequences, or matched sequences from non-trypanosomatid organisms. One sequence (TS3206) clustered consistently within the *T. brucei* clade, close to *T. congolense* and *T. brucei*. It would be interesting to gain more information on the trypanosome that yielded this sequence, given its close phylogenetic relationship to trypanosomes of economic importance as human and livestock pathogens. Five sequences were identified that sat within the trypanosomatids but outside the *T. brucei* clade. These sequences do not closely match any existing sequences and phylogenetic trees did not give sufficient resolution to draw firm conclusions regarding their identity. Further work is necessary to identify these sequences, for example using other genetic regions with reference sequences available such as glycosomal glyceraldehyde phosphate dehydrogenase or small subunit ribosomal genes for further identification [52]. Three sequences matched sequences from non-trypanosomatid organisms – *Dimastigella trypanoformis*, *Malassezia restricta* and an uncultured fungus, and phylogenetic analysis confirmed that four further sequences which did not match any existing sequences also sat outside the trypanosomatids.

### Implications for ITS primers

Diagnostic PCRs based on the ITS region rely on interspecies variation in sequence length to identify trypanosome species and subspecies [23]. This study raises a number of concerns regarding this approach for identification of trypanosome species in wildlife without sequencing. Twelve sequences in this study represented non-target organisms; these varied in length and overlapped with sequence lengths described for other trypanosome species, so could not be differentiated from target organisms by size alone. In addition, the diverse sequences in the *T. vivax* clade varied in length from 594 to 654 bp, and overlapped with the sequence length of *T. godfreyi* (648–650 bp). ITS primers in wildlife may give equivocal results and further investigation is necessary to establish whether they could be used to reliably identify trypanosome species or subspecies in wildlife without sequencing PCR products. Since species-specific primers may not amplify all trypanosomes of interest, the use of ITS PCR primers followed by sequencing is a good approach to investigate diversity of trypanosome infections in wildlife, but could be combined with other genetic regions to give greater phylogenetic resolution.

### Conclusions

Analysis of the ITS region of trypanosomes circulating in wildlife in two distinct geographical areas identified a large number of trypanosome species, including species that had not been identified before in wildlife as well as a number of species that are of importance as livestock pathogens, and revealed new diversity within the *T. vivax* clade. Although wildlife has been recognised as a source of livestock pathogens for many years, the addition of phylogenetic information raises many questions regarding the trypanosomes of wildlife and livestock, particularly regarding transmission, host sharing and pathogenicity. However, the absence of reliable high-throughput diagnostic tools to identify trypanosomes in wildlife makes investigations difficult and further phylogenetic analysis is likely to be necessary to explore these complex relationships.

## References

[1] Adams ER, Hamilton PB, Gibson WC (2010) African trypanosomes: celebrating diversity. Trends in Parasitology 26: 324–328. 20382076 10.1016/j.pt.2010.03.003

[2] Majiwa PAO, Maina M, Waitumbi JN, Mihok S, Zweygarth E (1993) *Trypanosoma (Nannomonas) congolense* - molecular characterization of a new genotype from Tsavo, Kenya. Parasitology 106: 151–162. 8383313 10.1017/s0031182000074941

[3] McNamara JJ, Mohammed G, Gibson WC (1994) *Trypanosoma (Nannomonas) godfreyi* sp. nov. from tsetse flies in the Gambia - biological and biochemical characterization. Parasitology 109: 497–509. 7800418 10.1017/s0031182000080756

[4] Malele I, Craske L, Knight C, Ferris V, Njiru ZK, et al (2003) The use of specific and generic primers to identify trypanosome infections of wild tsetse flies in Tanzania by PCR. Infection, Genetics and Evolution 3: 271–279. 10.1016/s1567-1348(03)00090-x 14636688

[5] Hamilton PB, Adams ER, Malele II, Gibson WC (2008) A novel, high-throughput technique for species identification reveals a new species of tsetse-transmitted trypanosome related to the *Trypanosoma brucei* subgenus, Trypanozoon. Infection Genetics and Evolution 8: 26–33. 10.1016/j.meegid.2007.09.003 17964224

[6] Van den Bossche P, Chitanga S, Masumu J, Marcotty T, Delespaux V (2011) Virulence in *Trypanosoma congolense* Savannah subgroup. A comparison between strains and transmission cycles. Parasite Immunology 33: 456–460. 21204855 10.1111/j.1365-3024.2010.01277.x

[7] Ashcroft MT (1959) The importance of African wild animals as reservoirs of trypanosomiasis. East African Wildlife Journal 36: 289–297. 13672101

[8] Heisch RB, McMahon JP, Mansonbahr PEC (1958) The isolation of *Trypanosoma rhodesiense* from a bushbuck. British Medical Journal 2: 1203–1204. 13584899 10.1136/bmj.2.5106.1203 PMC2027234

[9] Okello AL, Gibbs EPJ, Vandersmissen A, Welburn SC (2011) One health and the neglected zoonoses: turning rhetoric into reality. Veterinary Record 169: 281–285. 21908565 10.1136/vr.d5378

[10] Geigy R, Kauffman M (1973) Sleeping sickness survey in the Serengeti area (Tanzania) 1971: I. Examination of large mammals for trypanosomes. Acta Tropica 30: 12–23. 4144952

[11] Picozzi K, Tilley A, Fevre EM, Coleman PG, Magona JW, et al (2002) The diagnosis of trypanosome infections: Applications of novel technology for reducing disease risk. African Journal of Biotechnology 1: 39–45.

[12] Mulla AF, Rickman LR (1988) How do African game animals control trypanosome infections? Parasitology Today 4: 352–354. 15463028 10.1016/0169-4758(88)90005-1

[13] Lehane MJ, Msangi AR, Whitaker CJ, Lehane SM (2000) Grouping of trypanosome species in mixed infections in *Glossina pallidipes* . Parasitology 120: 583–592. 10874721 10.1017/s0031182099005983

[14] Njiru ZK, Makum iJN, Okoth S, Ndungu JM, Gibson WC (2004) Identification of trypanosomes in *Glossina pallidipes* and *G. longipennis* in Kenya. Infection, Genetics and Evolution 4: 29–35. 10.1016/j.meegid.2003.11.004 15019587

[15] Rodrigues AC, Neves L, Garcia HA, Viola LB, Marcili A, et al (2008) Phylogenetic analysis of *Trypanosoma vivax* supports the separation of South American/West African from East African isolates and a new *T. vivax*-like genotype infecting a nyala antelope from Mozambique. Parasitology 135: 1317–1328. 18752705 10.1017/S0031182008004848

[16] Enyaru JC, Ouma JO, Malele II, Matovu E, Masiga DK (2010) Landmarks in the evolution of technologies for identifying trypanosomes in tsetse flies. Trends in Parasitology 26: 388–394. 20542733 10.1016/j.pt.2010.04.011

[17] Wastling SL, Welburn SC (2011) Diagnosis of human sleeping sickness: sense and sensitivity. Trends in Parasitology 27: 394–402. 21659003 10.1016/j.pt.2011.04.005

[18] Moser DR, Cook GA, Ochs DE, Bailey CP, McKane MR, et al (1989) Detection of *Trypanosoma congolense* and *Trypanosoma brucei* subspecies by DNA amplification using the Polymerase Chain Reaction. Parasitology 99: 57–66. 2797872 10.1017/s0031182000061023

[19] Majiwa PAO, Webster P (1987) A repetitite deoxyribonucleic-acid sequence distinguishes *Trypanosoma simiae* from *Trypanosome congolense* . Parasitology 95: 543–598. 2827093 10.1017/s0031182000057978

[20] Masiga DK, McNamara JJ, Gibson WC (1996) A repetitive DNA sequence specific for *Trypanosoma (Nannomonas) godfreyi* . Veterinary Parasitology 62: 27–33. 8638390 10.1016/0304-4017(95)00847-0

[21] Masiga DK, Smyth AJ, Hayes P, Bromidge TJ, Gibson WC (1992) Sensitive detection of trypanosomes in tsetse flies by DNA amplification. International Journal for Parasitology 22: 909–918. 1459784 10.1016/0020-7519(92)90047-o

[22] Masake RA, Majiwa PAO, Moloo SK, Makau JM, Njuguna JT, et al (1997) Sensitive and specific detection of *Trypanosoma vivax* using the polymerase chain reaction. Experimental Parasitology 85: 193–205. 9030669 10.1006/expr.1996.4124

[23] Cox A, Tilley A, McOdimba F, Fyfe J, Eisler M, et al (2005) A PCR based assay for detection and differentiation of African trypanosome species in blood. Experimental Parasitology 111: 24–29. 16054487 10.1016/j.exppara.2005.03.014

[24] Njiru ZK, Constantine CC, Guya S, Crowther J, Kiragu JM, et al (2005) The use of ITS1 rDNA PCR in detecting pathogenic African trypanosomes. Parasitology Research 95: 186–192. 15619129 10.1007/s00436-004-1267-5

[25] Adams E, Malele I, Msangi AR, Gibson W (2006) Trypanosome identification in wild tsetse populations in Tanzania using generic primers to amplify the ribosomal RNA ITS-1 region. Acta Tropica 100: 103–109. 17109808 10.1016/j.actatropica.2006.10.002

[26] Kaare MT, Picozzi K, Mlengeya T, Fevre EM, Mellau LS, et al (2007) Sleeping sickness - a re-emerging disease in the Serengeti? Travel Medicine and Infectious Disease 5: 117–124. 17298919 10.1016/j.tmaid.2006.01.014

[27] Mubanga J (2008) Animal trypanosomiasis in the Eastern Province of Zambia: epidemiology in the recently-settled areas and evaluation of a novel method for control. Edinburgh: University of Edinburgh.

[28] Anderson NE, Mubanga J, Fevre EM, Picozzi K, Eisler MC, et al (2011) Characterisation of the wildlife reservoir community for human and animal trypanosomiasis in the Luangwa Valley, Zambia. PLOS Neglected Tropical Diseases 5: e1211. 21713019 10.1371/journal.pntd.0001211 PMC3119639

[29] Ahmed HA, MacLeod ET, Hide G, Welburn SC, Picozzi K (2011) The best practice for preparation of samples from FTA cards for diagnosis of blood borne infections using African trypanosomes as a model system. Parasites and Vectors 4: 68. 21548975 10.1186/1756-3305-4-68 PMC3108913

[30] Hall TA (1999) BioEdit: a user-friendly biological sequence alignment editor and analysis program for Windows 95/98/NT. Nucleic Acids Symposium Series 41: 95–98.

[31] Thompson JD, Higgins DG, Gibson TJ (1994) Clustal-W-Improving the sensitivity of progressive multiple sequence alignment through sequence weighting, position-specific gap penalties and weight matrix choice. Nucleic Acids Research 22: 4673–4680. 7984417 10.1093/nar/22.22.4673 PMC308517

[32] Stevens JR, Noyes HA, Schofield CJ, Gibson W (2001) The molecular evolution of Trypanosomatidae. Advances in Parasitolog 48: 1–56. 10.1016/s0065-308x(01)48003-1 11013754

[33] Drummond AJ, Ashton B, Buxton S, Cheung M, Cooper A, et al. (2010) Genious v5.3 Available from http://www.geneious.com.

[34] Hasegawa M, Kishino H, Yano TA (1985) Dating of the human ape splitting by a molecular clock of mitochondrial DNA. Journal of Molecular Evolution 22: 160–174. 3934395 10.1007/BF02101694

[35] Swofford DL (2003) PAUP* Phylogenetic Analysis Using Parsimony (*and Other Methods). Version 4 ed. Sunderland, Massachusetts: Sinauer Associates.

[36] Cortez AP, Ventura RM, Rodrigues AC, Batista JS, Paiva F, et al (2006) The taxonomic and phylogenetic relationships of *Trypanosoma vivax* from South America and Africa. Parasitology 133: 159–169. 16650339 10.1017/S0031182006000254

[37] Hamilton PB, Stevens JR, Gaunt MW, Gidley J, Gibson WC (2004) Trypanosomes are monophyletic: evidence from genes for glyceraldehyde phosphate dehydrogenase and small subunit ribosomal RNA. International Journal for Parasitology 34: 1393–1404. 15542100 10.1016/j.ijpara.2004.08.011

[38] Haag J, O'hUigin C, Overath P (1998) The molecular phylogeny of trypanosomes: evidence for an early divergence of the Salivaria. Molecular and Biochemical Parasitology 91: 37–49. 9574924 10.1016/s0166-6851(97)00185-0

[39] Stevens JR, Noyes H, Dover GA, Gibson WC (1999) The ancient and divergent origins of the human pathogenic trypanosomes, *Trypanosoma brucei* and *T. cruzi* . Parasitology 118: 107–116. 10070668 10.1017/s0031182098003473

[40] Gibson WC, Stevens JR, Mwendia CMT, Makumi JN, Ngotho JM, et al (2001) Unravelling the phylogenetic relationships of African trypanosomes of suids. Parasitology 122: 625–631. 11444615 10.1017/s0031182001007880

[41] Urakawa T, Majiwa PAO (2001) Physical and transcriptional organization of the ribosomal RNA genes of the savannah-type *Trypanosoma congolense* . Parasitology Research 87: 431–438. 11411940 10.1007/s004360100383

[42] Gashumba JK, Baker RD, Godfrey DG (1988) *Trypanosoma congolense* - the distribution of enzymic variants in East African and West Africa. Parasitology 96: 475–486. 3405634 10.1017/s0031182000080112

[43] Adams ER, Hamilton PB, Rodrigues AC, Malele II, Delespaux V, et al (2010) New *Trypanosoma (Duttonella) vivax* genotypes from tsetse flies in East Africa. Parasitology 137: 641–650. 19961657 10.1017/S0031182009991508

[44] Magona JW, Mayende JSP, Olaho-Mukani W, Coleman PG, Jonsson NN, et al (2003) A comparative study on the clinical, parasitological and molecular diagnosis of bovine trypanosomosis in Uganda. Onderstepoort Journal of Veterinary Research 70: 213–218. 14621317

[45] Lefrancois T, Solano P, Bauer B, Kabore I, Toure S, et al (1999) Polyerase chain reaction characterisation of trypanosomes in *Glossina morsitans submorsitans* and *G. tachinoides* collected on the game ranch of Nazinga, Burkina Faso. Acta Tropica 72: 65–77. 9924962 10.1016/s0001-706x(98)00080-1

[46] Fasogbon AI, Knowles G, Gardiner PR (1990) A comparison of the isoenzymes of *Trypanosoma (Duttonella) vivax* isolates from East Africa and West Africa. International Journal for Parasitology 20: 389–394. 2358323 10.1016/0020-7519(90)90156-h

[47] Dirie MF, Murphy NB, Gardiner PR (1993) DNA fingerprinting of *Trypanosoma vivax* isolates rapidly identifies intraspecific relationships. Journal of Eukaryotic Microbiology 40: 132–134. 8461886 10.1111/j.1550-7408.1993.tb04892.x

[48] Morlais I, Ravel S, Grebaut P, Dumas V, Cuny G (2001) New molecular marker for *Trypanosoma (Duttonella) vivax* identification. Acta Tropica 80: 207–213. 11700177 10.1016/s0001-706x(01)00160-7

[49] Rodrigues AC, Paiva F, Campaner M, Stevens JR, Noyes HA, et al (2006) Phylogeny of *Trypanosoma* (Megatrypanum) *theileri* and related trypanosomes reveals lineages of isolates associated with artiodactyl hosts diverging on SSU and ITS ribosomal sequences. Parasitology 132: 215–224. 16197590 10.1017/S0031182005008929

[50] Weitz B (1963) The feeding habits of *Glossina* . Bulletin of the World Health Organization 28: 711–729. 13999790 PMC2554947

[51] Vale GA (1974) Responses of tsetse flies (Diptera, Glossinidae) to mobile and stationary baits. Bulletin of Entomological Research 64: 545–588.

[52] Hamilton PB, Gibson WC, Stevens JR (2007) Patterns of co-evolution between trypanosomes and their hosts deduced from ribosomal RNA and protein-coding gene phylogenies. Molecular Phylogenetics and Evolution 44: 15–25. 17513135 10.1016/j.ympev.2007.03.023

[53] Hoare CA (1970) Systematic description of the mammalian trypanosomes of Africa. In: Mulligan HW, editor. The African Trypanosomiases. London: George Allen and Unwin.

[54] Stevens JR, Brisse S (2004) Systematics of trypanosomes of medical and veterinary importance. In: Maudlin I, Holmes PH, Miles MA, editors. The Trypanosomiases. Wallingford, Oxfordshire: CABI Publishing.

[55] Dillmann JSS, Townsend AJ (1979) Trypanosomiasis survey of wild animals in the Luangwa Valley, Zambia. Acta Tropica 36: 349–356. 44099

[56] Claxton JR, Faye JA, Rawlings P (1992) Trypanosome infections in warthogs (*Phacochoerus aethiopicus*) in the Gambia. Veterinary Parasitology 41: 179–187. 1502780 10.1016/0304-4017(92)90077-m

[57] Zweygarth E, Mihok S, Majiwa PAO, Kaminsky R (1994) A new *Nannomonas*-type trypanosome: Isolation, in vitro cultivation and partial characterisation [abstract]. Malaysian Society of Parasitology and Tropical Medicine. Kuala Lumpur.

